# Different Conceptions of Mental Illness: Consequences for the Association with Patients^†^

**DOI:** 10.3389/fpsyg.2013.00269

**Published:** 2013-05-15

**Authors:** Hanfried Helmchen

**Affiliations:** ^1^Department of Psychiatry and Psychotherapy, Campus Benjamin Franklin, Charité – University Medicine BerlinBerlin, Germany

**Keywords:** conceptions of mental illness, absolutization of partial conceptions, social space of the family, instrumentalization of the ill, social hygiene, preference diagnosis, normative content of human experience, bio-psycho-social model

## Abstract

Whenever partial knowledge is considered absolute and turned into ideological and dogmatic conceptions, the risk increases that the conditions for the people involved might become dangerous. This will be illustrated by casuistic examples of consequences of one-sided psychiatric conceptions such as social, biological, and psychological ideas about the treatment and care of the mentally ill. Present perspectives of an integrative model, i.e., an advanced bio-psycho-social conception about evidence-based characteristics on the social, psychological, and molecular-genetic level, require that all of these dimensions should be considered in order to personalize and thereby improve the care and treatment of the mentally ill.

The development of psychiatry has been accompanied by the debate about various conceptions of mental illness and the prevailing of one or the other of them at different times. Different consequences on attitudes in treating and caring for mentally ill patients of always one prevailing conception has been the stronger the more the conception focused only on a partial aspect of mental illness. This will be illustrated by casuistic examples

1. for the relationship of conception to practice
2. for the consequences of (at least of one-sided) conceptions on individual patients
3. Finally in contrast to such absolutization of partial conceptions (“Verabsolutierung von Partialerkenntnissen” (Jaspers, 1913) an integrative concept will be suggested.

## Conception and Practice

The German epigonal placarding of the controversy between Heinroth^1^ (and Ideler^2^) on the one hand and Jacobi^3^ as well as Nasse^4^ on the other hand as one between “psychicists” and “somaticists” in the first half of the nineteenth century disregards the fact that these representatives of different conceptions of mental illness in practice had more in common than was separating them (Kutzer, 2003). Both reasoned rationally with the conception of excitability developed by the Scottish physician John Brown (“Brownianism”) – his thesis of excitability dominated medicine at that time – and planned their treatment with mechanical coercion measures as “contrastimulation” (Schott and Tölle, 2006).

My opinion is that the practice of treatment and care of the mentally ill depends less upon a disease conception but more upon the experience, attitude, and personality of the psychiatrist – at least in his individual development.

However, this does not mean that there is no influence from the commonly accepted conceptions of the time. Thus the psychicist- versus somaticist-controversy may also indicate how much the reception of conceptions depends upon the connotations of the respective epoch. Today both terms are used in an almost opposite meaning: whereas psychicists such as Heinroth – at that time in the tradition of the Romantic – ascribed mental illness to the emotions of guilt about a sinful and failed life and reasoned with this a treatment with mechanical (“somatic”) coercion measures as a pedagogic therapy that currently is considered inhumane; but today the psychicists are misunderstood as representatives of a morally positively seen psychological-psychotherapeutic medicine. Conversely, at that time “somaticists” were psychiatrists who – such as Griesinger^5^ – ascribed mental illness to brain diseases and thereby were seen as more modern and humane, whereas nowadays “biological” psychiatrists are criticized as biological reductionists. However in the first half of the nineteenth century the introduction of a somatic conception of mental illness was a great step forward in the direction of appreciation of the mentally ill as ill persons [therefore it is no surprise that Griesinger also supported psychotherapy (Tölle, 2002)]. Today further important aspects of these conceptions are recognized: the risks of passivity and dependence of the medical-somatic disease conception, which protects the ill, and guilt in the disease conception of the psychicists, which also indicates self-responsibility.

## Unconditionality of Partial Conceptions

Even if psychiatric disease conceptions are attenuated or changed by medical experience, they can develop considerable effects, particularly if they do not grasp the complexity of mental illness but only a partial aspect of it and if this is then accentuated dogmatically. This is the case especially with persons outside of psychiatry who know the world of the acting medical persons only indirectly or only by hearsay. This will be made clear by three concepts or clusters of concepts: that of social psychiatry, that of biological psychiatry, and that of psychological medicine respectively psychotherapy.

### Social psychiatry

In the 1960s social conditions and consequences of mental illness increasingly came to the fore with young psychiatrists. In the UK – as the cult movie “Family Life” suggested – the social space of the family was seen as pathogenic; mental illness was understood as a reaction to a morbid society; or mental illness was even asserted to be a fiction of psychiatrists, most valuably by the Hungarian-American psychiatrist Szasz (1961).

In Italy the unbearable conditions of accommodation or custody of the mentally ill in large psychiatric hospitals, such as in Görz and Triest in northern Italy, caused the psychiatrist Basaglia (1968) to “liberate” these mentally ill by “negation,” i.e., to urge the closure of these large hospitals and, thanks to successful political exertion of influence, to realize this with the law number 180 in 1978.

This forcible and radical reform in Italy led to the disadvantage of many severely mentally ill persons and their helplessly overburdened families, who had to take in their otherwise not cared-for ill family members, and not before, with the development of community mental health centers, the basic idea of extramural-rehabilitative support of the mentally ill gained acceptance (Pycha et al., 2011). Still, the older history of social psychiatry shows that such ideological excess of a right basic idea leads to instrumentalization of the ill and finally to inhumanity.

The term “social psychiatry” appeared in the beginning of the twentieth century (Ilberg, 1904) in the context of terms such as “social pathology” or “social hygiene” as a rational reasoning for governmental efforts to control the social conditions and consequences of mental illness (e.g., syphilis, alcoholism, “asocial” psychopathy, vagabondage) by social, particularly even eugenic measures (Grotjahn, 1912; Rüdin, 1931; Priebe and Finzen, 2002; Schmiedebach and Priebe, 2003). During the economic misery after World War I these aims of social psychiatry were radicalized by the eugenic and thereby biological ideas of “racial hygiene” all the way to “euthanasia” (Schmiedebach and Priebe, 2003).

This process of convergence, even merging social psychiatry into “racial hygiene” caused other, much older forms of philanthropically or economically motivated forms such as “family care” or “open care” as social support systems for the mentally ill outside the asylums to fade into the background and narrowed them to modes of social control of the mentally ill. This development became terribly clear with the “reform” psychiatrists Paul Nitsche and Valentin Faltlhauser, who stipulated in the 1920s to bring the mentally ill out from hospital custody and to support them extramurally (Nitsche, 1931) but then in the 1930s, in the context of increasing ideologization in the interest of the collective (the “people”) advocated the social control of the mentally ill and finally murdered them during the war.

After World War I Faltlhauser became a close associate of Gustav Kolb. With his conception of “open care” Kolb initiated an internationally recognized psychiatric reform. The conception of “open care” was based upon outpatient care and a social support network for the chronically mentally ill. As a senior staff member in the psychiatric hospital of Erlangen Faltlhauser also took over the position of a care physician (“Fürsorgearzt”). Finally he was one of the leading reform psychiatrists and in 1929 he became the director of the Kaufbeuren psychiatric hospital where he also established “open care.” Together with Kolb and Hans Roemer^6^ he published “Die offene Fürsorge in der Psychiatrie und ihren Grenzgebieten” (“Open Care in Psychiatry and its Related Areas”) in 1927. Even in 1932, in his textbook of psychiatric care, he recommended the treatment of the chronically ill and rejected euthanasia measures. However, Faltlhauser pursued from the beginning the elimination of so-called “psychopaths”:

“…One of the most difficult questions of the treatment of psychopaths in open care is the question of marriage of psychopaths. It is not too much to assert that 80% of psychopaths marry a psychopath. It is the obligation of social care to prevent such an intended marriage as far as possible … (Because even) tireless information (is useless), perhaps the suggestion of incapacitation might be successful” (Roemer et al., 1927).

In contrast the current conception of social psychiatry, developed after World War II, is indeed also extramural but most notably oriented to the individual by helping the chronically mentally ill in a graded system of institutional aids to lead a more or less self-determined life in society^7^.

My opinion is that partial conceptions of mental illness might indeed convey transiently less recognized aspects to the public awareness. However, the more selective they are, the more they let other aspects be forgotten, and the more they become dogmatic, the more they can become dangerous for the individual ill person in practice.

This will be illustrated by three examples from the practice:

1. A young assistant in the psychiatric hospital, convinced of social psychiatry, refused to take over a patient with an acutely delirious state from surgery because he “was somatically ill.” (At that time this seemed for me to be a special form of brainless psychiatry).
2. At the height of the cult movie “Family Life” young colleagues implicitly addressed reproaches to the parents, mainly those of patients with schizophrenia, of bearing the blame for the manifestation of the disease – although this of course elevated contratherapeutically the emotional level of tension in the family.
3. A student with schizophrenia, decompensated during her university examinations, developed a postpsychotic residual state that was not accepted by her young therapist. The therapist intensively urged the patient to participate in an active rehabilitation program, which the patient tried to avoid. Several weeks later the patient committed suicide outside the hospital. Presumably she felt overburdened by the program.

### Biological psychiatry

The impressive improvement of the treatment of mentally ill that was made possible by the development of psychotropic drugs 60 years ago led the practice of drug treatment and research to the neurochemistry of the brain and the development of new drugs. For this focus on the brain and thereby on the biological foundations of mental illness the term “biological” psychiatry has become established.

However currently this term seems to be fading and to be substituted by the term “neuroscientific” after the possibility of gaining knowledge by the various neuroimaging measures developed during the last 30 years broadened research on the neuronal determinants of psychic functions and diseases so that psychiatry currently is neuroscientifically oriented. From a psychiatric viewpoint this research is disease-oriented brain research and its counterpart in practice is among others a differentiated drug treatment as well as neuropsychologically based methods of behavioral therapy.

Along with this objectifying, quantifying, disease-oriented view there is the risk that the patient’s feeling of illness will fade into the background and be recognized only insufficiently, i.e., that the patient’s experience of changes of his inner world as well as of his capacity to acting, his processing of his disease, his awareness of disease-conditioned disturbances of his relationship to his environment will pass from view. These different perspectives of disease versus feeling ill of the mentally ill person will be illustrated by my own experience:

- In the 1950s in a large outpatient clinic for people with epilepsy I tried to relieve patients from their seizures. My emphasis was on the disease. Side effects of the necessary drug treatment had to be accepted by the patient. With increasing experience my view widened from the disease to the feeling of being ill on the part of the patient. Today the patient is not only informed about the side effects of the treatment but, as appropriate, it will be decided together with the patient for which therapy objective he is willing to accept which burdens [this development was recently named “preference diagnosis” (Mulley et al., 2012)].

My opinion: The objectifying narrowing view of the disease leads to the disadvantage of an empathetic assessment of the patient’s feeling of being ill. This becomes comprehensible when biomedical research publications almost exclusively speak about research *on* patients, whereas it should be termed research *with* patients, because the individual subject should be invited to participate in a research intervention but it should not be researched on him as an object.

### Psychotherapy

During the past decades the concept of psychoanalytic psychotherapy has been joined or even opposed by many other conceptions of psychotherapy. This has led in practice – due to “the closeness of this field of the art of healing to a space free of sanctions” (Ritschl, 1989) – to the increase of private modifications of conceptions and finally – perhaps according to the contemporary “postmodern” credo of some philosophers that “everything goes” (Feyerabend, 1983) – in a few cases resulted in deadly quackery. Perhaps as a counter-reaction some therapists tried to assert rigorously the conception of the method that they performed, i.e., to keep it “pure.” Also the standardization of therapy manuals has been promoted.

My opinion is that not only the training in a specific method of psychotherapy and the indication for a specific state of mental disease determine the choice of a certain treatment in an actual case but also the normative content of the human experience of the therapist.

This can be seen in the establishment of therapy objectives – not only in psychodynamic but also in other psychotherapies such as, e.g., in deconditioning (sometimes even manipulating) techniques of behavioral therapy. “Is it about adaptation, optimal adaptation to the social environment, such as if the meaning of human life is classification or relationship to others? Or is the objective the maximal evolvement of the patient’s potential such as if the criteria of a healthy existence are only inside the single individual?” (Ritschl, 1989).

However if psychotherapy – in this case the psychoanalytic kind as recently published by the philosopher Paul Biegler (2011) – is declared dogmatically as the ethical imperative in order to support the autonomy of depressive patients, then danger threatens. This became apparent when a patient with depression sued his psychotherapist for withholding him antidepressant drugs (Klerman, 1990), or a patient with schizophrenia asked in court for compensation because for years his psychotherapist had refused to treat him with drugs.

## An Integrative Conception

Against the narrowing of conceptions that depict only partial aspects of mental illness and whose ideological radicalization during the past century had disastrous consequences for the mentally ill, most psychiatrists today follow a bio-psycho-social concept. This concept is reasoned in the experience of psychiatrists who see in their everyday practice how much the mental state of their patients is influenced by interaction with their social environment as well as by earlier impressions in the microsocial space of the family. These latter ones may be of developmental psychological or of biological-genetical nature.

In the 1960s the psychiatrist Hans-Joachim Bochnik introduced a graphical scheme in his Frankfurt University Hospital – the “Bochnik Triangle” – in which the grade of expression of somatic, psychic, and social influence on the current state of disease was to be mirrored (Bochnik et al., 1967). Thus the assistants had to turn their attention systematically toward all of these determining dimensions of being mentally ill. The conception became internationally well-known as the bio-psycho-social model by the Science-publication of Engel (1977). However, it has been criticized for being arbitrary and vague with regard to causal explanations of mental and behavioral disorders, and no rule exists for weighing the relevance of the various conceptions (Ghaemi, 2009). Therefore, the arbitrariness of the model should be reduced by focusing on scientifically proven concepts, to be tested for empirical evidence in the individual case, and taken as provisional in a longitudinal perspective (Brendel, 2003). Nevertheless, it may be helpful in two directions: at first, it should be used as a didactic tool to direct the psychiatrist toward a systematic exploration of the patient’s intern and extern context because the knowledge gained by this procedure may be helpful to guide the patient in overcoming his being ill; such preliminary trials to assess the complex texture of the disease at the clinical macro-level can deepened today at the micro-level: at second, the model encourages the psychiatrist to open his mind for real interdisciplinary considerations of the causal interchange between social, psychic, and neurobiological determinants of mental disorders which is hoped to be developed in the future as some stimulating findings indicate.

Thus, e.g., the brain researcher Florian Holsboer recently reported an example for such a gene-environment interaction. Holsboer et al could confirm the hypothesis that defined variations of the gene for the protein FKBP5, which modulates the function of the corticoid receptor, the most important receptor of the hormonal stress system, makes the bearer particularly sensitive for trauma expositions, which initiate a depression (Zimmermann et al., 2011).

This example, as one of to-date numerous others (Caspi et al., 2002; Haddad and Meyer-Lindenberg, 2012), substantiates the hope of recognizing genetic risk profiles for interactions with specific environmental somatic, psychic, and social factors for individuals and thereby to approach the objective of individualized medicine.

However, if this individualization is focused on its somatic base, i.e., to be content with the question which drug is the right one for which patient, and if by that loses sight of the psychic and social context, the chance will be lost to move from individualizing to personalizing medicine, medicine that recognizes the patient as a person in all of his relationships and does justice for him.

My final opinion concludes that disease conceptions are effective as background coordinates for the practicing psychiatrist. They help him to organize the complex diversity of phenomena: they are instrumental in nature. Even partial conceptions can help to provide particular awareness for a less considered but important aspect. However they become dangerous if they are not understood only as instruments to be used only transiently but are made ideologically absolute under the influence of dominating ideas in societal context, if they increasingly shut out important parts of reality, and if they instrumentalize the ill person – whether for scientific, or for political, or for personal purposes.

## Conflict of Interest Statement

The authors declare that the research was conducted in the absence of any commercial or financial relationships that could be construed as a potential conflict of interest.

## Footnotes

^1^Heinroth, Johann August Christian (1773–1843), founder of the worldwide first Chair of Psychiatry at the University of Leipzig (1811).

^2^Ideler, Karl (1795–1860), head of the “lunatic asylum” at the Charité in Berlin.

^3^Jacobi, Maximilian (1775–1858), founder of the mental hospital in Siegburg, near Bonn.

^4^Nasse, Friedrich (1778–1851), Bonn.

^5^Griesinger, Wilhelm (1817–1868), chair of psychiatry at the Berlin Charité 1865–1868.

^6^Hans Roemer was a convinced representative of the eugenic prevention of mental illnesses (including forced sterilization) but – as opposed to Faltlhauser – rejected euthanasia by a clear memorandum and retired ahead of time 1940 as director of the psychiatric hospital Illenau when he saw that he was unable to stop the deportation of his patients (Roelcke, 1993, 2012).

^7^This indicates also a change of the meaning of terms with the times going, i.e., the term of social psychiatry comprises a cluster of fairly different conceptions. Nevertheless, all conceptions are at risk to become absolutized.
